# Developing global measurement guidance to strengthen adolescent well-being data generation and use at country level

**DOI:** 10.1093/heapol/czaf106

**Published:** 2026-09-30

**Authors:** Sarah C Keogh, Holly Newby, Titus Divala, Parviz Abduvahobov, Sarah Baird, Tapas Chatterjee, Julianna Deardorff, Evelyne Degraff, Renée de Jong, Sithembile Dlamini-Nqeketo, Danielle Engel, Tamrat Gebremichael, Priya Karna, Jonathan D Klein, Constance Mackworth-Young, Terrance Magoro, Abdullah Al Mamun, Jose Marquez, Hlamalani Queen Nenzhelele, Mily Ramirez Ruiz, Jennifer Requejo, Zoya Ali Rizvi, Anshu Mohan, Regina Guthold

**Affiliations:** Maternal, Newborn, Child and Adolescent Health and Ageing Department, World Health Organization, 20 Avenue Appia, Geneva 1211, Switzerland; Maternal, Newborn, Child and Adolescent Health and Ageing Department, World Health Organization, 20 Avenue Appia, Geneva 1211, Switzerland; Maternal, Newborn, Child and Adolescent Health and Ageing Department, World Health Organization, 20 Avenue Appia, Geneva 1211, Switzerland; Division for Peace and Sustainable Development, Health and Education Section, Education Sector, United Nations Educational, Scientific and Cultural Organization, 7 Pl. de Fontenoy-UNESCO, Paris 75007, France; Department of Global Health, Milken Institute School of Public Health, George Washington University, 950 New Hampshire Ave NW, 4th floor, Washington, DC 20037, United States; WHO Country Office for India, Office of the WHO Representative to India, 537, A Wing, Nirman Bhawan, Maulana Azad Road, New Delhi 110 011, India; School of Public Health, University of California, Berkeley, 2121 Berkeley Way, Berkeley, CA 94704, United States; Organización Panamericana de la Salud, Carrera 9 No. 76–49 Piso 5 oficina 501 - 502, Edificio El Nogal, Bogotá D.C., Colombia; Partnership for Maternal Newborn and Child Health, 20 Avenue Appia, Geneva 1211, Switzerland; WHO Country Office for South Africa, 351 Francis Baard St, Pretoria Central, Pretoria 0002, South Africa; United Nations Population Fund, 730 Third Avenue, New York, NY 10017, United States; Plan Canada, 245 Eglinton Ave E #300, Toronto, ON M4P 0B3, Canada; WHO Country Office for India, Office of the WHO Representative to India, 537, A Wing, Nirman Bhawan, Maulana Azad Road, New Delhi 110 011, India; Department of Pediatrics, Adolescent Medicine, Stanford University, 750 Welch Rd, Suite 210, Palo Alto, CA 94304, United States; London School of Hygiene and Tropical Medicine and Biomedical Research and Training Institute, 10 Seagrave Road, Avondale, Harare, Zimbabwe; WHO Country Office for South Africa, 351 Francis Baard St, Pretoria Central, Pretoria 0002, South Africa; Poche Centre for Indigenous Health, Faculty of Health, Medicine and Behavioural Sciences, The University of Queensland, 74 High St, Brisbane, QLD 4066, Australia; Wellbeing Research Centre, Harris Manchester College, University of Oxford, Mansfield Rd, Oxford OX1 3TD, United Kingdom; National Department of Health, Dr. AB Xuma Building, 1112 Voortrekker Road, Pretoria Townlands 351-JR, Pretoria 0187, South Africa; Ministerio de Salud y Protección Social, Carrera 13 32-76, Bogota D.C. 110311, Colombia; Global Financing Facility for Women, Children and Adolescents, World Bank Group, 1818 H Street NW, Washington, DC 20433, United States; Department of International Health, Johns Hopkins Bloomberg School of Public Health, 615 N. Wolfe Street, Baltimore, MD 21205, United States; Adolescent Health and Nutrition Division, Ministry of Health & Family Welfare, Government of India, Nirman Bhawan, New Delhi 110011, India; Partnership for Maternal Newborn and Child Health, 20 Avenue Appia, Geneva 1211, Switzerland; Maternal, Newborn, Child and Adolescent Health and Ageing Department, World Health Organization, 20 Avenue Appia, Geneva 1211, Switzerland

**Keywords:** adolescent health, well-being, adolescent data, measurement, global guidance

## Abstract

Adolescence is a critical life stage marked by rapid physical, cognitive, and social development with implications for well-being in adulthood and of subsequent generations. Although global attention to adolescent well-being has grown over the past decade, efforts to measure it remain fragmented, sector specific, and include a greater number of indicators of risks and negative outcomes compared to positive well-being. To address these challenges, the World Health Organization, in collaboration with the Partnership for Maternal, Newborn and Child Health and other United Nations (UN) partners, developed new measurement guidance to support countries in assessing adolescent well-being in a holistic, multisectoral way across the five domains of the UN’s consensus Adolescent Well-being Framework (AWF) focused on health and nutrition, connectedness, safety, learning, and agency and resilience. This article describes the process and results of the guidance development. The process incorporated adolescent and youth perspectives, was supported by a multidisciplinary Expert Consultative Group and included several steps, starting with operationalizing the AWF for measurement. Draft guidance was then developed, and the feasibility of its implementation was assessed in Colombia, India, and South Africa. Feedback from country stakeholders led to substantial revisions—clarifying linkages to the broader measurement landscape, strengthening implementation information, and improving usability—before finalization. The guidance supports countries to identify and assemble existing data across all domains of the AWF, identify data gaps, assess current government-led efforts to improve adolescent well-being, and translate findings into action. Rather than prescribing new indicators or data collection efforts, it provides a structured approach and an Excel-based data tool to improve the use of existing data. By promoting a practical, country-led approach and building on existing systems, the guidance aims to support governments in strengthening insight, accountability, and action for adolescent well-being.

Key messages

- New guidance developed by the World Health Organization and other UN agencies supports countries in assessing adolescent health and well-being across all domains of the Adolescent Well-being Framework, including compiling existing data, assessing government-led actions, and translating evidence into action.
- The guidance responds to documented limitations in the measurement landscape—including fragmented efforts, lack of holistic assessment, preponderance of negative outcomes, and underuse of existing data—by laying out a practical, adaptable approach for countries to coordinate across sectors and meaningfully engage adolescents and youth.
- The content and structure of the guidance were shaped by adolescent perspectives, expert group input and feedback from country stakeholders.
- Countries will need to address potential implementation challenges, including identifying and taking steps to fill remaining data gaps, building multisector collaboration, engaging adolescents throughout the process, and ensuring dedicated time, resources and political capital.

## Introduction

Adolescence, spanning 10–19 years of age, is a time of rapid physical, cognitive, social, and emotional development and a critical period to lay the foundation for well-being (WHO 2025a). Experiences during this period profoundly influence health and well-being throughout the life course and across generations. Adolescent health has been a recognized priority on the international agenda for decades, as exemplified by the International Conference on Population and Development Programme of Action adopted in 1994, which dedicated a specific chapter to addressing the needs and rights of adolescents (UNFPA 2004). Since 2015, as adolescents have been highlighted as central to the sustainable development agenda (United Nations 2018, Alfvén et al. 2019), there has been increased global attention and accumulating evidence around their health and well-being. For example, the 2016 Lancet Commission on Adolescent Health and Wellbeing advanced understanding of this critical period by compiling global data and synthesizing evidence across different sectors (Patton et al. 2016), and the Second Lancet Commission provided extended analysis and a call for action (Baird et al. 2025). The importance of adolescent well-being has been reflected in numerous sector-specific strategies (Joerger and Hoffmann 2002, OECD 2017, 2021, UNICEF 2022), in particular the Global Accelerated Action for the Health of Adolescents (AA-HA!), an inter-agency guidance document which provides countries with a structured, multisectoral roadmap to strengthen policy and programming for adolescent health and well-being (WHO 2023). Major global advocacy events, such as the 2019 Adolescent Call to Action (Major Group for Children and Youth 2019) and the 2023 Global Forum for Adolescents (PMNCH 2023b), have called for increased investment in adolescent well-being, meaningful adolescent engagement, and the need for more guidance, in particular around measurement, to support better evidence-based policies and programming. The newly developed measurement guidance described in this paper is intended as a contribution to filling this gap.

The Adolescent Well-being Framework (AWF), developed by the United Nations (UN) H6+ partner agencies in 2020, provides a consensus definition of adolescent well-being and a holistic conceptual framework to guide multisectoral policies and programmes. Organized around five interlinked domains (good health and optimum nutrition; connectedness, positive values and contribution to society; safety and a supportive environment; learning, competence, education, skills, and employability; and agency and resilience), it defines adolescent well-being as a state where ‘adolescents thrive and are able to achieve their full potential’ (Ross et al. 2020). The AWF reflects the breadth of well-being as a concept, encompassing both subjective (e.g. adolescents’ own evaluations) and objective (e.g. material conditions) aspects, as well as both protective (e.g. safe environment) and risk factors (e.g. substance use).

In the light of the developments of the past decade, the importance of a holistic approach to measuring adolescent well-being has become increasingly clear—to monitor progress, support accountability, and inform decision-making in a comprehensive and multisectoral way. This has been further highlighted in dialogues with regions and countries (PMNCH 2022b) and by international organizations working at the country level (Abduvahobov et al. 2024). To support countries in monitoring adolescent health in a standardized way, in 2024 the Global Action for the Measurement of Adolescent Health (GAMA), an advisory group to the World Health Organization (WHO) and other UN agencies established in 2018, recommended a set of 47 priority indicators (WHO 2024a). While the GAMA-recommended indicators span a range of topical areas, including determinants such as poverty and education, they focus primarily on physical health and risk factors, with limited coverage of positive well-being indicators, and do not comprehensively cover the five domains of the AWF.

Summaries of the current measurement landscape have argued that relevant data remain fragmented, with existing measurement efforts, including global accountability initiatives such as the Sustainable Development Goals, generally prioritizing distinct aspects of adolescent well-being or outcomes within specific sectors, such as mental ill health, education, or risky behaviours like alcohol or tobacco use (Newby et al. 2022, Guthold et al. 2023). There are additional barriers to obtaining a comprehensive picture of adolescents’ lives, including the fact that data collection often does not span the entire adolescent age range of 10–19 years, in part due to the unique challenges of collecting data on young adolescents (Way 2014), and that people’s understanding of well-being can vary across cultures and contexts (Newby et al. 2022). Notably, global measurement and accountability initiatives and multi-topic survey programmes lack questions on adolescents’ own subjective perspectives (Guthold et al. 2021).

Taken together, these issues highlight several key challenges: first, the need to bring together existing data across sectors and domains; second, the importance of generating and using data that reflect the complexity of well-being, including its breadth, subjectivity, and contextual variability, and not just objective measures of poor health or risk factors; and third, how to better incorporate adolescents both as data providers and also as collaborators who shape processes around data collection and use. To address these challenges, the WHO, in collaboration with the Partnership for Maternal, Newborn and Child Health (PMNCH) and other UN partners, has developed new guidance to support countries in identifying, compiling and using existing data on adolescent well-being across the five domains of the AWF, as well as assessing relevant government actions, using both existing indicators recommended by GAMA and newly developed metrics to assess multisectoral efforts (Guthold et al. 2023). This guidance aims to help countries coordinate across sectors to compile existing data, identify data gaps, engage adolescents and other key stakeholders in their interpretation, and translate data into multisectoral action. This manuscript describes the development process and resulting guidance. The aim is to present the rationale, content, and potential value for countries implementing the approach.

## Methods

### Overview of the guidance development process

The development of the measurement guidance was overseen by a WHO/PMNCH coordinating team. To ensure the guidance was based on the latest science and given the multidimensionality and complexity of the AWF domains, the coordinating team convened a time-bound Expert Consultative Group. This group consisted of 28 academics and other professionals deliberatively selected through a competitive call to cover multiple disciplines (including anthropology, economics, education, psychology, public health, and sociology), with measurement expertise across all AWF domains, and representing different regions, gender balance, and inclusion of early-career experts under the age of 30 (Guthold et al. 2023). The Expert Consultative Group defined and actively contributed to the development process through structured consultations, including virtual meetings, document reviews, and collaborative decision-making on key elements of the guidance. The process involved a number of steps (depicted in Fig. 1 and explained in more detail in the following sections), starting with operationalizing the AWF for measurement, developing draft guidance, assessing its feasibility in three countries (Colombia, India, and South Africa), and refining and finalizing it in light of feedback.

**Figure 1.**
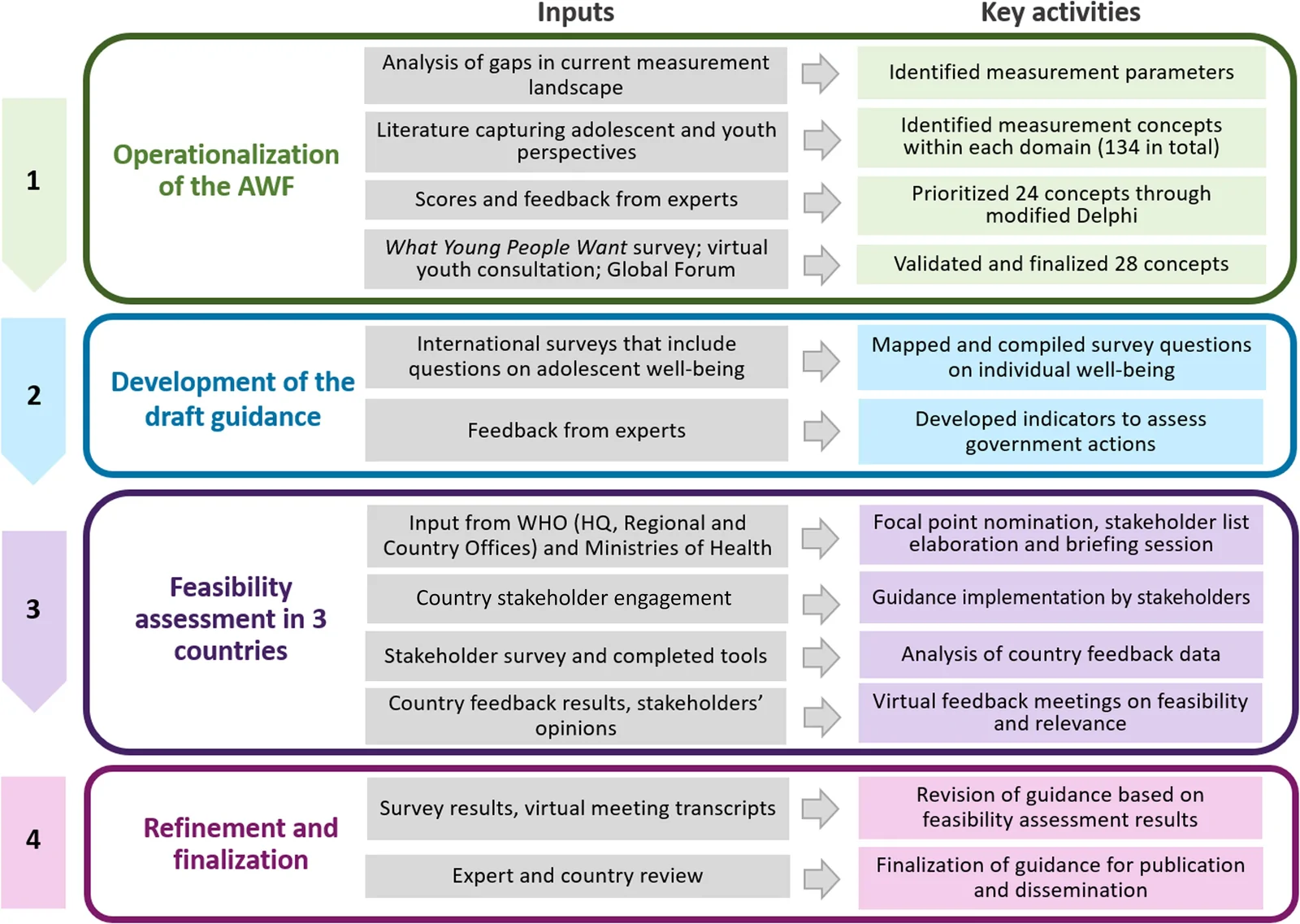
Steps in developing the guidance.

### Operationalization of the adolescent well-being framework

#### Measurement parameters

The Expert Consultative Group identified two complementary priority areas that countries should focus on to measure their progress in improving adolescent well-being: (i) individual well-being, to assess outcomes experienced by adolescents themselves and (ii) government actions, to assess the extent to which systems, policies, and programmes are in place and effectively implemented to improve individual outcomes. These two areas were selected to allow countries not only to track the lived realities of adolescents but also to evaluate the institutional efforts and mutisectoral responses that influence those realities. The Expert Consultative Group and coordinating team then agreed on a set of measurement parameters, as follows:

- Focus on quantitative measures in order to facilitate monitoring and cross-country dialogue. Where available, qualitative data can be used to complement, contextualize, and enrich understanding of observed patterns.
- Include subjective measures, as this represents a major evidence gap for adolescent well-being at the global level: while both objective and subjective measures are integral to a holistic understanding of adolescent well-being, existing indicator sets are heavily weighted towards objective measures (Newby et al. 2022).
- Prioritize positive measures, such as belief in ability to reach learning goals, given their underrepresentation in the field and their critical importance in determining ‘well-being’ (rather than ‘ill-being’): although measurement should capture both positive and negative aspects, commonly used indicators are dominated by more negative measures such as substance use, high-risk sex, bullying, anxiety, and depression (Guthold et al. 2021).
- Prioritize use of existing data and systems over new data collection: countries often have unused data on adolescent well-being or have systems in place that could be strengthened to collect more relevant, disaggregated, and actionable data; prioritizing these will enable greater efficiency and harmonization with ongoing measurement efforts.
- Ensure guidance is standardized (to facilitate implementation, technical support, and comparability across countries) yet flexible enough to be relevant and adaptable to diverse and rapidly changing cultural and political contexts and different and evolving understandings of well-being.
- Enable assessment of the well-being of ‘all’ adolescents across the wide range of developmental stages between the ages of 10 and 19 and across subpopulations (e.g. gender, sexuality, school attendance, marital and childbearing status, socio-economic status, disability, religion, race/ethnicity, exposure to conflict, trauma, and other vulnerabilities), through allowing disaggregation to capture these varied adolescent experiences.

#### Identification, prioritization, and finalization of key concepts for measurement

To identify key aspects of individual well-being across the five AWF domains, the process began with a rapid literature review with particular attention to adolescent perspectives (Fattore et al. 2019, Casas 2022, Renwick et al. 2022, Hossain et al. 2023), followed by consultation with the Expert Consultative Group. This resulted in a long list of 137 potential concepts for measurement. The term ‘concept’ is used as a broad designation for aspects of well-being that are at a more conceptual level and have yet to be narrowed down and operationalized for measurement; in other words, each concept encompasses multiple potential indicators and accommodates various ways of measuring it. This long list of concepts was then refined through a modified Delphi exercise (Hsu and Sandford 2007, Trevelyan and Robinson 2015), to systematically prioritize concepts. Expert Consultative Group members rated each concept out of 10 for its relevance to adolescent well-being; based on the relatively high scores across all concepts, those with a median score of ≥8 and a mean of ≥7 were retained, yielding 40 concepts. These were further consolidated to eliminate overlap, resulting in a shortlist of 24 concepts. Youth validation played a critical role in finalizing the list, based on three sets of inputs: (i) the What Young People Want global survey (PMNCH 2023c); (ii) a virtual consultation in August 2023 with six diverse youth constituencies (recruited through the PMNCH Adolescent and Youth Constituency; PMNCH 2023a) between ages 18 and 30, representing five of the six WHO regions and focused on a range of issues including LGBTQ+, disabilities, empowerment, and sexual and reproductive health and rights; and (iii) feedback during the Global Forum for Adolescents (PMNCH 2023b). These inputs led to the refinement and expansion of the list, culminating in 28 prioritized concepts.

### Development of the draft guidance

#### Mapping of standardized international survey questions onto prioritized concepts for individual well-being

To facilitate the identification of country-level data relevant to the prioritized concepts for measuring individual well-being, major standardized international surveys that touched on adolescent well-being were inventoried. To be included, surveys had to be conducted in more than one world region, include adolescents (at least part of the 10–19 age range) in their respondent sample, and ask questions related to at least two AWF domains. The inventoried surveys included household surveys (such as the Multiple Indicator Cluster Surveys; UNICEF 2024), school-based surveys (such as the Health Behaviour in School-aged Children study; HBSC 2017), as well as adolescent-focused studies such as the Global Early Adolescent Study (GEAS 2025) (Fig. 2). Relevant questions from the standard questionnaires of the selected instruments were mapped onto the prioritized concepts in order to create a ‘menu’ of questions for each concept.

**Figure 2.**
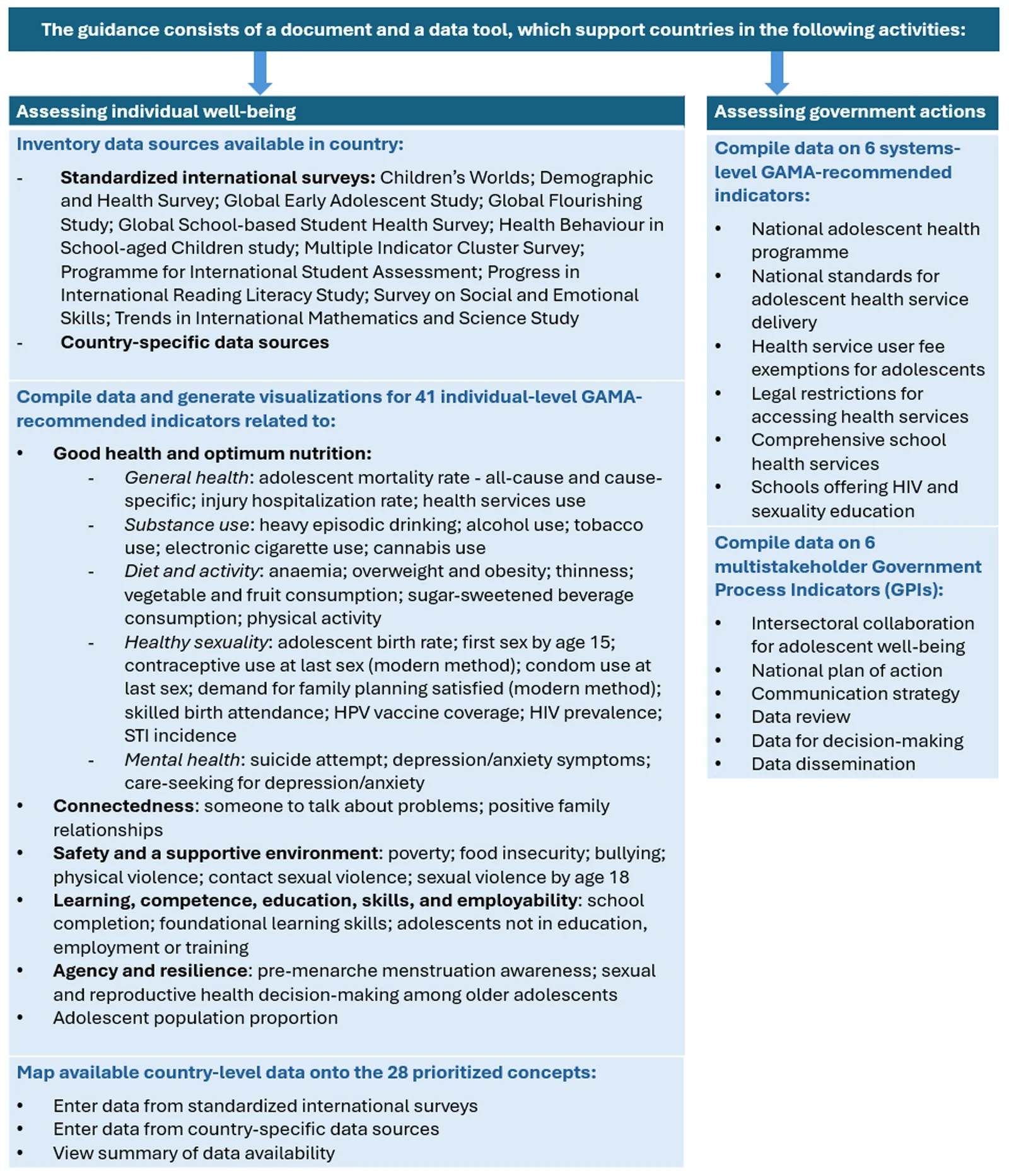
Activities supported by the guidance.

#### Developing indicators to assess government actions

In parallel to the development of individual well-being measurement guidance, the coordinating team developed a set of process indicators to assess government-led actions to support adolescent well-being. The first step was to establish the parameters of the indicators, with a decision to focus on government-led, multisectoral actions in order to complement existing sector-specific input and process indicators and reflect broader system-level commitment and coordination. The second step was to review key resources related to adolescent well-being in order to identify relevant, measurable government actions. Resources with relevant government actions included the logical framework of the AA-HA! (WHO 2023) and a set of principles and processes recommended to governments making commitments to support adolescent well-being programming in advance of the Global Forum for Adolescents (PMNCH 2022a). The third step was to translate those actions into indicators by reviewing examples of input, process, and output (Papanicolas et al. 2022, Schultz et al. 2024) metrics and ensuring alignment with the principles of meaningful adolescent and youth engagement (PMNCH 2020). This resulted in six draft government process indicators that were then reviewed by the Expert Consultative Group and refined based on their suggestions.

#### Draft guidance document and data tool

The elements described above formed the core of the draft measurement guidance, which consisted of a document containing background information and implementation guidance and a set of two draft Excel-based tools: one to compile data on individual well-being from standardized international surveys and the other to assess government actions using the process indicators.

### Assessing the feasibility of implementing the measurement guidance at the country level

The relevance and usability of the draft measurement guidance was then tested as part of a feasibility assessment in three countries (Colombia, India, and South Africa) selected to represent different regions, with large cohorts of adolescents and strong commitments to adolescent well-being. The assessment protocol was submitted to the WHO Research Ethics Review Committee and received a waiver (protocol ID: ERC.0004068). The WHO/PMNCH coordinating team worked with the WHO Regional and Country Offices to secure support from relevant ministries and appoint a focal person in charge of compiling a stakeholder list and organizing a series of meetings. In each country, between 17 and 50 stakeholders were invited from the various ministries and sectors involved in adolescent well-being. These included ministries of health, education, social affairs, youth, justice, community development, gender (women and child development), and transport; national statistics offices, civil society organizations, academia, bilateral organizations, and programme implementers, including three to five representatives from diverse youth organizations. Stakeholders had to be at least 18 years of age.

In each country, the assessment followed the same steps. After an initial virtual briefing session on the assessment, stakeholders took 2–4 weeks to implement the guidance, which consisted in jointly reviewing the guidance document and completing the Excel-based tools. Halfway through that implementation period, the coordinating team organized another informational session with stakeholders to clarify any emerging questions. Stakeholders then completed an individual online survey via LimeSurvey (LimeSurvey GmbH 2025) (subject to their informed consent) to gather their opinions on the relevance, usability (clarity and user-friendliness) and feasibility of implementing the guidance in their country, using 5-point Likert scales and open-ended questions. Each stakeholder was assigned an anonymous identification number.

Country feedback data were analysed using a mixed-methods approach. Quantitative survey results were imported into Excel for analysis, and responses were summarized using descriptive statistics to identify patterns across countries. Aggregate scores for dimensions such as relevance and feasibility were derived for each country for different parts of the guidance. Qualitative data from open-ended survey questions and meeting transcripts were thematically analysed to identify key challenges, recommendations, and country-specific contextual factors. The analysis framework focused on three dimensions: relevance, usability, and feasibility of implementation, with particular attention to cross-cutting themes that emerged across multiple countries. The completed tools were also reviewed for potential gaps and errors in understanding. The coordinating team then organized between one and three meetings in each country to present and discuss preliminary results with all participating stakeholders; transcripts from these meetings also contributed to the qualitative analysis. The assessments were staggered in 2024, with India’s assessment running from April to July, South Africa’s from July to September, and Colombia’s from August to October. All consultations were held virtually and were recorded subject to the consent of all present.

### Refinement and finalization of the guidance

Findings from the three countries were synthesized to inform revisions to the guidance, as described in the results. The revised guidance was then shared with the Expert Consultative Group and the three countries for their feedback and validation before finalization.

## Results

This section describes four key results: a set of prioritized concepts recommended for individual well-being measurement, a set of government process indicators, the findings from the country feasibility assessment (which was based on draft guidance materials), and a description of the resulting final guidance for countries.

### Prioritized concepts for individual well-being measurement

The prioritization process resulted in a final validated list of 28 concepts, with between 4 and 8 in each AWF domain (Table 1). Specific changes and additions as a result of young people’s input are indicated in bold italics. In particular, young people pointed out the importance not only of having enough food but also of eating a balanced diet, given the rise of obesity among adolescents in several world regions. Regarding sexuality, they cautioned that programmes still lean mostly towards preventing negative outcomes and suggested more attention should be placed on positive aspects such as pleasure and enjoying stigma-free healthy sexuality. Regarding the importance of having support, they challenged the original wording ‘Having a trusted adult’, noting that often adolescents, especially LGBTQ+ youth, may not feel they have any adults that they can trust but may have trusted peer networks that fulfil that role. Young people also suggested three additions in response to perceived gaps and misalignments between domain names and their content: in Domain 2, being civically and socially active was suggested as an important requisite for the underrepresented aspect ‘contribution to society’, while in Domain 5, which focused mostly on agency, ‘adaptive capacity/resourcefulness’ was suggested to fill the gap around resilience. Similarly, in Domain 4, which focused mainly on education, young people brainstormed a concept to represent what is important for their well-being with regards to employability.

**Table 1.** Prioritized concepts for each adolescent well-being framework domain.

Adolescent well-being framework domain	Prioritized concepts
Domain 1: Good health and optimum nutrition	Adequate ***and balanced*** diet
	Adequate physical activity
	Good sleep
	***Healthy*** sexuality
	Life satisfaction
	***Overall health self-assessment*** ^a^
	Overall mental health self-assessment^a^
	Overall physical health self-assessment^a^
Domain 2: Connectedness, positive values and contribution to society	***Civically and socially active***
	Having at least one trusted ***person***
	Positive relationships (in general)
	Positive relationships with parents/family
	Positive relationships with peers
	Sense of belonging, feeling accepted, respected, and valued by others
	Positive values
Domain 3: Safety and a supportive environment	Freedom from all types of violence
	Sense of safety in day-to-day life
	Sense of being treated equitably/free from discrimination
	Supportive environment
Domain 4: Learning, competence, education, skills and employability	Belief in ability to reach learning goals (self-efficacy)
	Social and interpersonal skills
	Sense of belonging to school/work
	***Confidence in their skills and ability to earn a living when time comes***
Domain 5: Agency and resilience	Hope/optimism/confidence about the future
	Self-esteem/self-worth
	Sense of agency and self-efficacy
	Sense of purpose/meaning
	***Adaptive capacity/resourcefulness***

***Bold italics*** indicate changes and additions as a result of young people’s input.

^a^These were independently prioritized as important and measurable concepts. For example, there are a number of widely used questions, such as ‘How do you describe your health in general?’, that would fall under ‘Overall health self-assessment’.

### Indicators on government-led multisectoral efforts

A set of six government process indicators aimed at assessing multisectoral, whole-of-government actions to improve adolescent well-being were developed (Table 2). Each indicator was composed of three to four characteristics designed to be rated on a four-point scale ranging from ‘not addressed’ to ‘fully addressed’. Instructions on scoring and cut-offs were provided in the draft data tool.

**Table 2.** Government process indicators.

Indicator	Characteristics
1. Intersectoral collaboration for adolescent well-being	Coordination across government ministries and agencies for the promotion of adolescent well-being, characterized by: adolescent well-being holistically addressed, participation of multiple ministries, regularly scheduled meetings/activities, meaningful adolescent and youth engagement.
2. National plan of action	Existence of a national action plan with targets on adolescent well-being, characterized by: adolescent well-being holistically addressed, resources for plan, time-bound targets, alignment with national development priorities.
3. Communication strategy	Well-being-related communication strategy(ies) informing and empowering adolescents on their rights and relevant services, characterized by: participation of multiple ministries, multiple channels of communication, ongoing, nationwide implementation, meaningful adolescent and youth engagement.
4. Data review	Disaggregated, nationally representative data on adolescent well-being available, characterized by: data collected directly from adolescents, data available for each of the five adolescent well-being domains, intersectoral data availability and quality audit.
5. Data for decision-making	Available data related to adolescent well-being are jointly reviewed and used to support decision-making, characterized by: multisector, multistakeholder involvement, data regularly reviewed, demonstrated contribution to decision-making, meaningful adolescent and youth engagement.
6. Data dissemination	Data made available to relevant stakeholders—including adolescents and their communities, characterized by: timely dissemination, targeting of key non-technical audiences, multiple channels of communication, meaningful adolescent and youth engagement.

### Feasibility assessment of the draft guidance

The draft measurement guidance used in the feasibility assessment consisted of a document with background information and two Excel-based tools (one to compile data on individual well-being and the other to assess government actions). The feasibility assessment results are based on stakeholder feedback gathered through two means: (i) individual online survey results [8 questionnaires from Colombia (24% response rate), 23 questionnaires from India (46% response rate), and 7 from South Africa (41% response rate)], and (ii) inputs from participants during the feedback meetings, which were attended by 15 stakeholders in Colombia, 14 in India, and 13 in South Africa.

The survey results and the feedback meetings both showed that the participants found the guidance helpful in their countries. Stakeholders appreciated the focus on leveraging existing data and systems, especially when resources for additional data collection are limited, as well as identifying critical data gaps. In the survey, participants rated the government process indicators as highly relevant and found the exercise of filling in these indicators useful for identifying priorities at the national level. Table 3 presents a synthesis of key areas for improvement suggested by participants, together with the resulting refinements to the guidance materials. Panel 1 provides some illustrative quotes from feasibility assessment participants. Full results and summary scores from the feasibility assessment are available in Supplementary Appendix A.

**Table 3.** Summary of country feedback leading to guidance revisions.

Summary of country feedback	Incorporation of feedback into guidance
**Relevance of the guidance**	
Guidance needs to be better linked to existing measurement landscape and global initiatives. Information about the rationale for the guidance and the development of its components needs to be strengthened. It would be useful to include examples or case studies of countries that have implemented.	Provided additional background information and recentred the guidance around AA-HA! and GAMA. Information on the development of guidance added in an appendix. Panel added with an example from Madagascar.
**Usability of the guidance**	
Elaboration is needed on how to implement the guidance. Tools should be streamlined and user friendly to reduce the burden on countries. More detailed instructions on how to complete the tools are needed.	New chapter laying out the implementation steps developed. A single, combined tool with embedded instructions was created; it included the two separate draft tools as well as a previously separate tool for the compilation of GAMA indicators. Video tutorial on completing the tool was developed.
**Country adaptability**	
There must be greater flexibility in adapting the prioritized concepts to the local context as long as all five domains remain represented. It is important that the tool allows for data from country-specific studies and has information regarding the synthesis of data from different sources.	A box describing the prioritized concepts and how they may be adapted was added to the guidance document. A new function was added to the tool for inventorying data from country-specific sources, together with information on synthesizing data from different sources.
**Subnational level use**	
It is important to highlight that the guidance can be implemented at the subnational level and provide information on how to do that.	The option of implementing the guidance at subnational levels was highlighted.
**Multisectoral and multistakeholder collaboration**	
Efforts pertaining to adolescent well-being are often siloed, so the guidance needs more detail on the range of sectors and ministries that should be involved, as well as emphasis on non-governmental stakeholders and adolescents/youth.	Enhanced text around multisectoral and multistakeholder collaboration. Added an annex with an illustrative list of potential stakeholders. Added a panel on meaningful adolescent and youth engagement. A youth-oriented summary of the guidance was developed.

Panel 1. **Illustrative quotes from feasibility assessment participants**

Colombia: ‘I question if the measurement really manages to address all the elements and nuances of being an adolescent in the territories of South America. The Amazon alone has as many ways of being an adolescent as there are birds and flowers.’

India: ‘Implementation is at State level; hence ownership and information also have to be with them.’

South Africa: ‘We found the [government process] indicators very useful. They really helped us realize where we are and what needs to be done to work towards improving adolescent well-being.’

### Final guidance

The final guidance comprises two interlinked resources: a foundational document and an Excel-based data tool (WHO 2025b). The document provides the context and purpose and outlines a practical approach for assembling and using adolescent health and well-being data. It begins by situating the guidance within broader global efforts, describes its objectives and intended audience, and introduces the accompanying data tool. It also provides direction on its implementation, outlining how countries can translate data into policy and programme action. The data tool allows countries to identify, compile, and visualize existing individual-level data and to assess government-led policies and programmes relevant to adolescent health and well-being. The tool begins with an instructions sheet and a clickable roadmap to orient the user to the different activities to complete.

As stated previously, the guidance document and tool are structured around two key components: individual adolescent health and well-being, and government actions. Figure 2 lists the activities and associated indicators for each component. The individual component includes the following activities (each corresponding to separate sheets in the tool): inventorying available data sources, compiling data related to the 41 individual-level GAMA-recommended indicators, and mapping data from international surveys and country-specific data sources onto the 28 prioritized concepts. Data entered under these various activities in the tool are used to automatically generate visualizations summarizing available data on individual health and well-being. The document provides additional information to support the implementation of these activities, including suggestions on country adaptation, such as aligning activities with existing country processes.

For the second component, the guidance recommends a total of 12 indicators for assessment of government actions supportive of adolescent health and well-being. This includes the original six specially developed multisectoral government process indicators, as well as the six systems-level GAMA-recommended indicators. All 12 indicators are composed of multiple items, and the tool provides definitions and instructions for assessment. Automatically populated summary sheets in the data tool give a snapshot of overall progress on each set of indicators.

## Discussion

Advancing adolescent well-being requires not just robust data, but the ability to translate data into action—to identify priorities, shape budget-backed and evidence-based policies, and monitor programmes for effective advocacy and accountability. In response to the fragmented adolescent well-being measurement landscape (Guthold et al. 2021, Kågesten et al. 2024), this guidance offers a practical approach to bridging the numerous sector-specific efforts underway, identifying what data are already available and using them more strategically, acknowledging that every country is at a different stage but has the opportunity to improve adolescent well-being by using existing data.

### Strengths of the guidance and its development

This approach is designed so that countries can build on existing data systems that are contextually adapted, while also promoting harmonization across contexts. This dual benefit—efficiency at country level and alignment with a global conceptual framework and measurement recommendations—is a major strength of the guidance. Moreover, the act of compiling data, disaggregated by age and sex, across sectors, and domains can foster cross-ministerial dialogue and can catalyse broader systems thinking about adolescent well-being, as evidenced during the feasibility assessment. Thus, this guidance frames adolescent well-being as a multisectoral issue shaped by education, safety, agency, and inclusion—alongside health—and aims to provide a practical step towards utilizing existing data for integrated action as laid out by the AA-HA! (WHO 2023). Additionally, the guidance provides a structured approach to assessing government-led, multisector actions to provide insight into accomplishments and areas for improvement. By providing an overall framework that can be integrated into countries’ existing monitoring systems across different sectors, it has the potential to contribute to improved national policy coherence and accountability, a major theme in the 2019 Adolescent Call to Action (Major Group for Children and Youth 2019, PMNCH 2023b).

The importance of meaningful adolescent and youth engagement in the programming and monitoring of their well-being has been consistently highlighted both in recent global advocacy initiatives as well as by countries themselves (PMNCH 2020). What is often lacking is follow through. The development of this guidance incorporated adolescents and youth perspectives in several ways. First, the overall breadth and continuity of adolescent and youth engagement built on the broader advocacy initiative that encompassed the Call to Action, the Global Forum for Adolescents and the What Young People Want campaign, spearheaded by PMNCH (WHO 2024b), which the guidance is a part of. Second, the guidance was initially developed based on existing literature documenting adolescent and youth perspectives, respecting the wealth of data that have already been contributed by young people across time and space instead of performatively asking for new input (Baird et al. 2025, Nagata et al. 2025). Third, there were additional, specific efforts to further incorporate younger perspectives into the development of the guidance—for example, the inclusion of young researchers in the Expert Consultative Group and the consultation with youth representatives on the draft list of prioritized concepts.

Given the salience of culture and context to understanding well-being, the development of the guidance tapped into expertise from a broad range of countries. The Expert Consultative Group itself was composed of members from across the six WHO regions. Representatives from each of the WHO regional offices were invited to participate in meetings and also provided bilateral inputs at key stages of the process, contributing perspectives from their region. The feasibility assessment brought in valuable insights from Colombia, India, and South Africa, for example, on the need for flexibility around the concepts and their interpretation in different contexts, and the importance of integrating available country-specific data, particularly on marginalized groups who may not be systematically included in cross-country survey programmes. These insights substantially influenced the finalization of the guidance.

### Limitations of the guidance development process

Despite the care taken in its development, the guidance development process faced several limitations. The breadth and complexity of adolescent well-being made it difficult to achieve full consensus among the Expert Consultative Group. The initial selection of the long list of concepts for prioritization generated much debate around which types of concepts—such as subjective versus objective, or input versus outcomes, or individual versus societal—were most important to capture. Subsequently, in the modified Delphi exercise, some concepts were scored within a narrow interquartile range, while scores for other concepts had a much wider interquartile range, reflecting the varying levels of consensus around what concepts should be prioritized. This is illustrative of the broader challenge of developing global guidance on measuring a concept that is so contextually specific. The feasibility assessment, while critical to informing the finalization of the guidance, was conducted in only three countries and had limited participation from adolescents and youth, despite efforts to include them. While young people did contribute to the other guidance development activities, all participants were at least 18 years old, meaning that the perspectives of younger adolescents were not represented.

The formulation of the government process indicators presented a unique challenge, reflecting broader tensions in measuring governance across diverse political systems and administrative arrangements. The substantial variation in how adolescent well-being is governed—through centralized versus decentralized systems, government versus civil society leadership, and diverse financing mechanisms—required careful consideration to create indicators that could capture meaningful progress while remaining applicable across contexts. This resulted in some deliberate simplification and pairing down of the indicators. Indeed, a financing indicator drafted earlier in the process was ultimately dropped due to these sorts of concerns.

Similarly, to maintain usability across diverse contexts, the data tool was kept relatively simple and does not yet allow for more advanced disaggregation beyond age and sex or for longitudinal data analysis. A key reason for this is that questions about subjective experience are not always understood in the same way across cultural and linguistic contexts, or when questionnaires operationalize the same concept in different ways, which can make direct comparisons difficult (OECD 2013, Cummins 2018). Thus, as a first step, the tool is designed to provide a snapshot of the available data. These limitations may have implications when it comes to implementing the guidance in other countries, for example, related to institutional adoption and ownership. While efforts were made to ensure the guidance is adaptable to different country contexts and systems, further adaptation may be needed for settings with limited existing infrastructure for these activities, such as fragile and humanitarian contexts.

### Potential implementation challenges

The guidance attempts to address long-standing weaknesses in adolescent well-being measurement. However, moving from guidance to practice will require attention to a number of implementation challenges. First, the guidance’s emphasis on existing data needs to be weighed against the risks of reinforcing a false sense of completeness. The apparent availability of data in some areas may create the impression that the evidence base is sufficient, even when existing data are only loosely aligned with the prioritized concepts. Critical gaps persist, for example, around measurement of certain concepts such as self-worth and agency. Furthermore, data may not be fully representative of all adolescents, for example, data may be lacking for the youngest ages or for the most vulnerable and/or marginalized populations. Thus, continued efforts are needed not only to compile and use existing data more effectively, but also to identify and fill key data gaps to ensure a more comprehensive and equitable understanding of adolescent well-being.

Second, while the guidance emphasizes the importance of cross-sectoral collaboration and meaningful adolescent and youth engagement in data processes, these outcomes are not necessarily easy to achieve. As noted during the feasibility assessment, building and sustaining broad coalitions to take this work forward can be particularly difficult in contexts where adolescent well-being lacks a clear institutional ‘home’ and related efforts remain siloed. Implementers must take deliberate steps to engage throughout the process a diverse range of stakeholders and sectors, including adolescents themselves, which can be especially challenging when such collaborations are not already in place. However, the findings from the feasibility assessment confirmed that although identifying the right partners and convening them required substantial effort, the benefits of doing so were clear and could ideally drive collaboration between previously siloed sectors.

Third, while the guidance is designed to support more data use, its implementation may burden those leading the process, especially at the start. Government officials and other stakeholders who would likely be involved are already managing numerous competing priorities and data-related demands. Proper implementation and sustainability require dedicated time, resources, effort, and political capital to assemble a multisectoral team, ensure meaningful engagement of adolescents and youth, identify relevant data sources and compile data, and assess government actions. Although the initial phase may require considerable time and coordination, based on the feedback obtained during the feasibility assessment, countries that invest in this effort are likely to find that subsequent updates and use of the tool will be more streamlined, although it will certainly require ongoing resources and commitment.

### Looking ahead

This guidance is intended as a practical starting point that country stakeholders can use immediately, drawing on already available data and encouraging accountability by incorporating government process indicators. While it is a first step for advancing adolescent well-being measurement, there is still a long way to go. At the global level, as previously noted, some areas of adolescent well-being remain especially difficult to measure, with limited or inconsistent indicators and a lack of standardized data collection tools (Guthold et al. 2021). Further advancing measurement will require continued work to develop tools that are capable of capturing the full diversity of adolescent experiences, including those that substantially vary by age and by gender, as well as ensuring that key indicators are included in monitoring frameworks. Additional work is also needed to better track government inputs and processes, including financing indicators to better assess donor versus government expenditures. However, indicators and tools alone will not be sufficient—as articulated by country participants in the feasibility study, country capacity must be built to enable effective use of these tools and approaches, along with political buy-in to the importance of monitoring. By laying out a way forward for integrated monitoring and action around adolescent well-being, this guidance can act as a catalyst for national accountability and cross-sector coordination.

The newly published guidance is expected to support ongoing efforts aligned with broader adolescent well-being initiatives in countries to compile and make use of adolescent well-being data. Its release is particularly timely, given the 2nd Lancet Commission’s call for evidence-based advocacy and multisectoral actions (The Lancet 2025). Previous exercises using an earlier version of the tool—including only the GAMA-recommended indicators—have already helped inform planning and policy discussions in countries such as Madagascar and Burundi (WHO 2025b). As of May 2025, planning is underway to use the updated version of the guidance in additional countries, with implementation expected in the coming months.

## Conclusion

The development of the guidance was informed by country stakeholder perspectives, and the feasibility assessment confirmed earlier research showing that while many countries collect sector-specific data on adolescent well-being, most often they are not systematically brought together and fully utilized to inform action (Kågesten et al. 2024). The development of the guidance document, which seeks to address this gap, followed an inclusive multistakeholder collaborative model through an iterative, consultative process, laying out a globally relevant, standardized yet adaptable approach to bring data together from multiple sectors to assess adolescent well-being. This paper’s careful documentation of each step of this process provides a replicable roadmap for other global coalitions looking to develop measurement or monitoring guidance on other topics. Ultimately, the aim is for this guidance to support governments and partners in building a more comprehensive, multisectoral evidence base for adolescent well-being—one that can be used to inform advocacy, policy, and programming and to drive progress in the lives of adolescents around the world.

## Supplementary Material

czaf106_Supplementary_Data

## Data Availability

The data underlying this article are available in the article and in its online supplementary material.

## Reflexivity statement

As a global initiative, the development of this measurement guidance incorporates data and perspectives from a diversity of experts and stakeholders from multiple countries across all world regions and of different seniority, gender, and ethnic identity. Authors come from a wide range of academic, UN, government and programmatic backgrounds. Nine authors (38%) are based in the Global South, and 16 (67%) are women. We are committed to inclusive global collaboration in the development and implementation of this guidance.

## Disclaimer

Sarah Keogh, Holly Newby, Titus Divala, Tapas Chatterjee, Renée de Jong, Evelyne Degraff, Sithembile Dlamini-Nqeketo, Priya Karna, Terrance Magoro, Anshu Mohan, and Regina Guthold are employed by the World Health Organization. Parviz Abduvahobov is employed by the United Nations Educational, Scientific and Cultural Organization. Danielle Engel is employed by the United Nations Population Fund. The authors alone are responsible for the views expressed in this publication, which do not necessarily represent the decisions, policy, or views of the World Health Organization, the United Nations Educational, Scientific and Cultural Organization or the United Nations Population Fund. Jennifer Requejo is employed by the Global Financing Facility for Women, Children, and Adolescents, World Bank Group. The findings, interpretations, and conclusions expressed in this article are entirely those of the author(s). They do not necessarily reflect the views of The World Bank, its Board of Executive Directors, or the governments they represent.
