# Supplementary material for: Developing global measurement guidance to strengthen adolescent well-being data generation and use at country level
Source: Health Policy Plan. 2026 Sep 30;41(Suppl 2):ii57–68. doi: 10.1093/heapol/czaf106 (PMC13626659; doi:10.1093/heapol/czaf106)
Supplement: Supplementary file 1 [file czaf106_supplementary_data.docx]

**Supplementary appendix A: Detailed results from the three-country feasibility assessment**

The findings presented here are based on stakeholders’ feedback gathered through two methods: individual online survey results (8 questionnaires from Colombia (24% response rate), 23 questionnaires from India (46% response rate), and 7 from South Africa (41% response rate)), and inputs from participants during the feedback meetings, which were attended by 15 stakeholders in Colombia, 14 in India, and 13 in South Africa. All identified stakeholders were invited to participate in all activities, including the online survey and the feedback meetings. While there was considerable overlap in who completed the online survey and who attended the feedback meetings, some stakeholders participated in only one or some of the activities, hence the different sample sizes for each activity.

*Usability of the guidance*

The different parts of the guidance were found to be clear (Figure A1). Stakeholders requested additional background information on the relevance of the guidance and specific indicators to the global context, how they fit with other initiatives, and the process followed to develop the guidance. They suggested expanding guidelines to facilitate implementation, specifically around purposes and intended users of each part of the tool, roles and responsibilities of different stakeholders, expected results and timelines for undertaking different activities, frequency of tool updates, funding/resource requirements and capacity-building needed. Participants from all three countries mentioned the value of having concrete examples from other countries who had already implemented the guidance. Respondents also requested more detailed instructions on how to complete the tools, with a step-by-step video. They noted the importance of making the tools as simple as possible, and minimizing their number or combining them, since often countries are already juggling a number of tools. A regional adviser on adolescent health commented: *“In order for tools to get used by countries, they must be as simple as possible.”*

Source: stakeholder surveys in Colombia (n=8), India (n=23) and South Africa (n=7).

**Figure A1. Stakeholders’ mean ratings on clarity of guidance document (1=not at all clear, 5=very clear)**

Stakeholders conducted a practical test of the usability of the tools by completing them. Table A1 presents an illustrative example of India’s summary sheet for the individual well-being tool, showing just international surveys (noting that the exercise did not aim to exhaustively map all surveys in India). The overall count column provides the total number of available survey questions related to each concept. Under each survey is the number of questions pertaining to each concept in that particular survey. In India, of the seven international surveys mapped, those providing the greatest number of relevant questions were Children’s Worlds (48 questions) and the Global Early Adolescent Study (GEAS, with 41 questions). Very few prioritized concepts have no questions at all (in grey), while most concepts have at least one survey question available to measure it.

**Table A1: Coverage of prioritized concepts by surveys conducted in India***

***** Because the purpose of this feasibility assessment was to test and obtain feedback on the draft global guidance, Colombia, India-specific surveys such as the National Family Health Survey were not included.

**Abbreviations:** Children's Worlds: Children's Worlds Survey; DHS: Demographic and Health Survey; GEAS: Global Early Adolescent Study; GSHS: Global School-Based Student Health Survey (Core and Expanded questionnaires); GFS: Global Flourishing Study (and adolescent version: GFS-Adol); HBSC: Health Behaviour in School-aged Children study; MICS: Multiple indicator Cluster Survey

Respondents expressed some concerns around the clarity of the prioritised concepts used to guide the individual well-being data compilation. Some suggested adding broad definitions to guide countries in better understanding concepts such as “healthy sexuality” or “connectedness”, but it was subsequently agreed during the feedback meetings that concepts might mean different things in different contexts, and having definitions may restrict countries’ freedom to interpret the concepts as is most relevant to their context. Stakeholders pointed out that while subjective measures are important, they may be challenging to measure and link to interventions, thus highlighting the need for objective measures to complement them.

*Relevance of the guidance*

Participants stated that the individual well-being tool could be useful for various purposes (Figure A2). In terms of tracking well-being, it could be used to monitor trends and disparities through for example the creation of disaggregated national indicator dashboards. This would help consolidate fragmented data across departments. A respondent from South Africa explained: *"We do have data sporadically in various departments, however, it is not integrated. So that’s one thing if as a country we can have just one hub where we can have data that talks to adolescent issues and is available at the click of a button."* Stakeholders appreciated the tool’s focus on using existing data in contexts where resources for additional data collection are often limited. However, they also valued the tool’s ability, through comprehensively inventorying available data, to help identify critical gaps and generate discussion on how to fill them. In terms of policymaking and programming, the tool could facilitate needs assessments to help set priorities and identify areas for improvement and greater resource allocation. It may be used to evaluate the impacts of actions and identify successful interventions for potential scale-up, though establishing a direct correlation between programmes and outcomes was flagged as a potential barrier. The data compiled through the tool could also be used as the basis for advocacy briefs on priority topics.

*Note: In Colombia, no stakeholders responded on usefulness for their own work.*

Source: stakeholder surveys in Colombia (n=8), India (n=23) and South Africa (n=7).

**Figure A2. Stakeholders’ mean ratings of the usefulness of the well-being tool (0=not at all useful, 10=extremely useful)**

Stakeholders rated the Government Process Indicators (GPIs) as highly relevant for their country context, with ratings of 4.6/5 or above (Figure A3). As one South Africa participant put it, *“we found the [government process] indicators very useful. They really helped us realize where we are and what needs to be done to work towards improving adolescent well-being”.* Respondents highlighted a number of uses for the GPIs, including needs assessments, quarterly reporting, policy formulation and programme planning, and fostering collaboration across a broad range of stakeholders from government agencies to civil society. They found the exercise of filling in these indicators useful for identifying priorities at the national level as one participant from Colombia commented:

*“The exercise has been interesting to identify and make visible these priorities, but also to look at how they can be planned. For example, in the case of the health sector, how can this planning be improved to take into account these gaps that we have identified, especially in participation and access to information and communication for adolescents and young people.”*

Source: stakeholder surveys in Colombia (n=8), India (n=23) and South Africa (n=7).

**Figure A3. Stakeholders’ mean relevance score for each National Action Indicator (1=not relevant, 5=very relevant)**

*Emphasising country adaptability*

While participants valued how standardised guidance enabled better support to countries and between-country dialogue, they emphasised the importance of being able to adapt the guidance to reflect country-specific processes. They wanted more information on how to integrate the guidance into existing national information systems and processes, and in particular how to include data from country-specific sources in the individual well-being data tool. For example, participants from Colombia were concerned about how to ensure the tool gives adequate visibility to marginalised groups of adolescents, such as indigenous youth, out-of-school adolescents or those with disabilities. Stakeholders from all countries highlighted the importance of making use of the many relevant country-specific studies to avoid duplicating efforts, and starting with indicators that are already collected in existing routine systems. They suggested adding guidelines on compiling and analyzing data from these multiple sources and using relevant qualitative data to complement the quantitative data compiled through the tool.

Stakeholders consistently rated the 28 prioritized concepts as highly relevant for understanding adolescent well-being and guiding policies in their countries, with mean scores ranging from 3.9 to 5.0 out of 5. However, they suggested more emphasis on countries’ flexibility to adapt the list to their own context, while ensuring coverage of all 5 domains and without modifying the concepts themselves. It was suggested that countries could be guided to cover a minimum number of prioritised concepts in each domain. Respondents particularly valued the flexibility to add concepts considered necessary for understanding adolescent well-being in particular contexts. One participant from Colombia commented: “*I question if the measurement really manages to address all the elements and nuances of being an adolescent in the territories of South America. The Amazon alone has as many ways of being an adolescent as there are birds and flowers*.” Examples of concepts considered key by Colombian stakeholders that were not part of the prioritized list include self-image, substance use, virtual relationships, interaction with nature, recreation, collective well-being; cultures, arts and heritage, comprehensive sexuality education, and compassion.

*Using the guidance at subnational level*

Another recurrent theme across the three countries concerned the importance of being able to apply the guidance and tools at the subnational level. In India and Colombia, many measurement activities and government actions occur at the subnational rather than national level. While stakeholders felt that the individual well-being tool was flexible enough to be used at subnational level, they suggested adding more specific guidance on how to adapt it for subnational use to identify gaps and inequities. A Colombia participant warned that: “*If the data do not give relevant information on gaps between populations, policies and programming are going to be focused on the national aggregate, maintaining gaps and inequities. It would be 'action with harm’.”*

Many of the actions listed under the GPIs also tend to be decentralised. As a participant from India commented: “*Implementation is at State level, hence ownership and information also has to be with them”*. Respondents highlighted challenges of working at the subnational levels, which in many cases lack human and economic resources, in addition to barriers related to their geographical dispersion. Colombian stakeholders also mentioned the problem of high staff turnover, making it more difficult to monitor actions and leading to important initiatives being lost; however, they were hopeful that completing the GPIs at the subnational level could provide a more complete picture of ongoing actions and initiatives.

*The need for cross-sectoral collaboration*

The guidance and tools’ multisectoral scope was seen to enable countries to build a more holistic picture of adolescent needs, and the fact that it requires cross-sectoral collaboration was viewed by all three countries as an opportunity to break the silos in which ministries sometimes work, by coming together to define intersectoral actions. A South African respondent commented: *“While it was a bit painful to put the group together, it really helped us to work across sectors which is required when dealing with adolescent well-being.”* A respondent from India similarly commented: “*The exercise forced us to work with other sectors which was very important.”* While the health sector has traditionally led these types of efforts, there were calls to involve other sectors and ministries (such as Women and Child Development, or Youth) in coordination roles, since they serve the adolescent as a whole and may have a more comprehensive understanding of adolescent well-being, rather than focusing more narrowly on health, education or livelihoods. While South Africa has been implementing a more intersectoral approach to adolescent well-being, led by the Presidency, stakeholders saw it as a key priority to establish clear processes for coordinating data and actions across government departments: *"With such high level questions, there is potential to answer some questions very differently depending on the lens of who is answering, and process for synthesizing input from the group - how is consensus reached, ensuring everyone is heard and there is fair representation"* (South Africa stakeholder). India and Colombia participants suggested a review be made of what relevant initiatives, committees and structures already exist that may facilitate cross-sectoral collaboration in this area. In Colombia, various well-being initiatives are already in place at the subnational level that could be built upon.

Stakeholders across countries also insisted on the importance of involving non-governmental stakeholders, in particular adolescents and youth (although participants highlighted the challenge of true inclusivity around age, gender, socio-economic status, and isolated regions). Respondents suggested adding more detail on meaningful adolescent and youth engagement at each step in the process of data compilation and use, and requested materials in more user-friendly formats. Participants from India and Colombia offered relevant examples of recent initiatives that engaged young people in policy processes at local and national levels. To facilitate cross-sectoral collaboration, a South African stakeholder suggested conducting detailed network and partner mapping prior to completing the GPIs.

*Using data for action*

The majority of respondents found the process for how to use these data for action to be clear, with an average rating of 4.1-4.4 out of 5 (Figure A1). Nonetheless, a number of respondents called for further elaboration on steps to take to ensure optimal use of results, such as defining a roadmap based on the findings, organising meetings to reflect on the findings, broad dissemination to all sectors, creating pedagogical processes to facilitate data use by adolescents, and supporting subnational entities in using the findings so that they can benefit from them immediately in their programs. Stakeholders suggested adding case studies of how the data compiled through the tool might be presented and used.
